# Impact of floral and geographical origins on honey quality parameters in Saudi Arabian regions

**DOI:** 10.1038/s41598-024-59359-y

**Published:** 2024-04-15

**Authors:** Wed Mohammed Ali Alaerjani, Mohammed Elimam Ahamed Mohammed

**Affiliations:** Department of Chemistry, College of Science, King Khalid University, Abha, Saudi Arabia

**Keywords:** Free acidity, Sugar profile, Floral origin, Geographical origin, Biochemistry, Chemical biology, Chemistry

## Abstract

This article examined the effect of geographical (different climate conditions) and floral origins on some quality parameters of honey including the activity of diastase enzyme. Moreover, some non-quality parameters were investigated such as the pH, fructose, glucose, ratio of fructose/glucose and invertase. The honey samples were collected from Asir (cold climate) and Jazan (hot climate) regions at the southwestern part of Saudi Arabia. The geographical origin significantly affected the mean value moisture of the Acacia honey (p-value = 0.02), conductivity of the polyfloral honey (p-value = 0.03), sucrose of the Acacia honey (p-value = 0.02), diastase activity of the Acacia (p-value = 0.001), Ziziphus (p-value = 0.046) and polyfloral honey (p-value ≤ 0.001), fructose of the Acacia honey (p-value = 0.01), glucose of the Ziziphus honey (p-value = 0.03), fructose/ glucose ratio of the Ziziphus honey (p-value = 0.035), and invertase activity of the polyfloral honey (p-value ≤ 0.001). Regarding the effect of the floral origin of the honey from Asir region, the sucrose percentage of the Acacia honey was significantly more than that of the polyfloral honey (p- value = 0.003), the diastase activity of the Acacia honey was significantly more than its activity in the Ziziphus honey (p- value = 0.044), glucose percentage of the Ziziphus honey was significantly more the glucose percentage of the Acacia honey (p-value = 0.009) and the fructose/ glucose ratio of the Ziziphus honey was significantly more than that of the Acacia and polyforal honeys (p-value = 0.011 and p-value = 0.045, respectively). Concerning the significant effects of the floral origin on the quality parameters of the honey samples from Jazan region, the moisture of the Ziziphus honey was significantly increased when compared to the moisture of the Acacia honey (p-value = 0.038), the acidity of the polfloral honey was significantly more than the acidity of the Acacia honey (p-value = 0.049), the sum of fructose and glucose of the polyfloral honey was significantly increased compared to that of the Acacia honey (p-value = 0.015), the pH of the Ziziphus hiney was significantly more than the pH of the polyfloral honey (0.011) and the fructose of the polfloral honey was significantly more than that of the Acacia honey (p-value = 0.031). The effect of the geographical origin of the honey samples on their quality parameters depends on their floral origin and the effect of their floral origin differs according to their geographical origin. This article suggests considering collectively the geographical and floral origins effect when developing honey standards. However, the Codex standards for honey started considering this issue when it changed the standard concentration of HMF in honey from not more than 80–40 mg/Kg for honeys from cold climate and 80 mg/Kg for honeys from hot climates.

## Introduction

Honey is a natural sweet substance that is produced by honeybees depending on the nectar of plants, secretions of plants living parts or excretions of plant sucking insects (Hemiptera). Honey is well known by its nutritional and medicinal values due to its chemical composition. Majorly, the honey is composed of carbohydrates and water beside minor bioactive compounds such as the organic acids, phenolic compounds, flavonoids, proteins and short peptides, vitamins, minerals and enzymes^1,2^. International, regional and national honey standards are created to fight adulteration of honey through measuring some quality parameters. The quality parameters of honey include the moisture, conductivity, acidity, sugar profile (sum of fructose and glucose beside the sucrose), diastase, invertase (Germany and Turkey), proline (Germany, Poland and Turkey) and hydroxymethylfurfural (HMF). Moreover, the honey standards include the investigation of health threatening parameters like presence of heavy metals (lead and cadmium), pesticides and microbes^1,3–5^.

High water content of honey may be due to immature honey (early harvest) or because of adulteration or fraud. Honeys with more than 17% moisture content are susceptible to fermentation by different species of yeasts^6^. The moisture of the honey in the standards has three values according to the floral origin as follows; not more than 20%, 23% (Heather honey (Calluna) and 25% (baker’s honey from heather)^1,3,4^.

The Electrical conductivity (EC) reflects the electrolyte content of honey and it is used to differentiate between nectar and honeydew honeys. It is directly related to the honey ash content, acidity and dark color^7^. The EC value of the honey depends on the floral origin as follows; the honeydew and chestnut honeys are with not less than 0.8 mS/cm, other blossom honeys are with not more than 0.8 mS/cm and some honeys are exceptions such as the Strawberry tree (Arbutus unedo), Bell Heather (Erica), Eucalyptus, Lime (*Tilia* spp.), Ling, Heather (*Calluna vulgaris*) Manuka or Jelly bush (Leptospermum), Tea tree (*Melaleuca* spp.)^1,3,4^.

Acidity of honey is an indication for fermentation (conversion of sugars to acids), geographical origin, floral origin and harvest season^8^. The standard honey acidity value is 50 milliequivalent acid per kilogram but there are some exceptions such as the acidity of the bakers honey which is not more than 80 milliequivalent acid per kilogram and the honey *Acacia tortilis* and *Acacia ehnbergiana* which are normally with high value of acidity if they are monofloral (with more than 50% Acacia pollens)^1,3,4^.

The honey quality parameters include measuring fructose, glucose and sucrose. The total fructose and glucose should be not less than 60% (60 g/100 g) except the honeydew honey and the mix of honeydew and blossom which contains not less 45%. Regarding the sucrose percentage of the honey, the lavender (*Lavandula* spp.) and borage (*Borago officinalis*) honeys contain not more than 15%. The honeys of false Acacia (*Robinia pseudoacacia*), Alfalfa (*Medicago sativa*), Menzies Banksia (*Banksia menziesii*), French honeysuckle (Hedysarum), red gum (*Eucalyptus camadulensis*), leatherwood (*Eucryphia lucida*, *Eucryphia milliganii*), *Citrus* spp., *Ziziphus* spp. and Acacia tortilis are characterized by not more than 10% sucrose. The other honeys contain not more than 5% of sucrose^1,3,4^. Sugars concentration in honey indicates its floral origin, maturity, adulteration by sugar syrups and prolonged feeding of honeybees by sucrose syrup^7,8^.

Fructose/glucose ratio is not considered as a honey quality parameter. It is used to predict the tendency of a honey sample to crystalize. The ratio of fructose/glucose was reported to be between 0.4 and 1.6 or more^9^.

Enzymes occur naturally in honey in small amounts, which is one of the most important characteristics that distinguish honey from other sweeteners. Honey enzymes participate in its ripening process through the conversion of the nectar and plant secretions or plant sucking insects excretions to mature honey^10^. The sources of honey enzymes vary from the bees themselves, nectar or pollen of the plant and they can be originated from microorganisms in honey such as the yeasts and bacteria. The most famous and prominent enzymes in honey are the diastase, invertase, glucose oxidase, catalase, serine proteases (pepsin, trypsin and chymotrypsin), acid phosphatase and dihydroxyacetone phosphatase^10–13^.

The diastase (α-amylase) is one of the most important enzymes in honey. It converts the amylose of nectar to maltose and glucose. It is considered as a main quality control factor for assessing honey freshness and overheating. The origin of diastase enzyme is attributed to the salivary secretions of bees^14,15^.

Invertase is an enzyme responsible for converting the sucrose to fructose and glucose to make a very concentrated solution of sugars which help in resisting honey fermentation. The activity of the invertase is an important parameter as indicator of honey freshness and thermal treatment. It is a better indicator than diastase because this enzyme is more sensitive to temperature and storage conditions than the diastase. The invertase activity is included in the honey standards of some European countries such as Germany, Poland, Turkey, Spain and Belgium. The minimum activity of invertase is 64 U/kg and values below this threshold are considered of low invertase activity^5,16–19^.

The physicochemical properties including the quality parameters of honey are affected by the floral origin, geographical and climate conditions, honeybees associated factors such as their nutrition, health and species and honey associated factors like its maturation, harvesting, processing, storage and the relations between its chemical constituents^20^.

This article investigated the collective impact of the floral origin and geographical origin (different climate conditions and altitude) on some quality and non-quality parameters of honey. We are trying to prove that the effects of the floral and geographical origins should be taken collectively and not separately. The investigated honey quality parameters were the moisture, conductivity, acidity, sum of fructose and glucose, sucrose and diastase activity. Moreover, some non-quality parameters were measured including the pH, fructose, glucose, fructose/ glucose ratio and invertase activity.

## Results

### Confirmation of the floral origin of the studied honey samples

**Figure 1:**
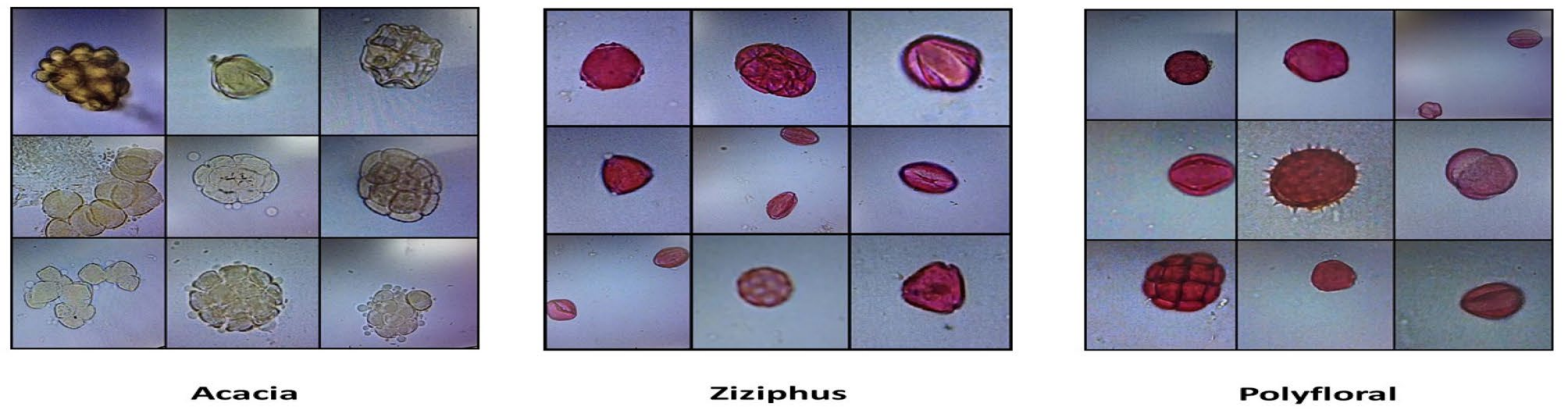
Representative result of the pollen analysis of the honey samples of this study.

As mentioned before, the floral origin was confirmed microscopically as Acacia, Ziziphus and polyforal honey samples as shown in Fig. 1.

### Effect of the geographical origin on the quality parameters of the honey samples

The mean, standard deviation, minimum and maximum values of the studied parameters of the honey samples are presented in Table1. The geographical origin significantly affected the moisture of the Acacia honey, conductivity of the polyfloral honey, fructose of the Acacia honey, glucose of the Ziziphus honey, sucrose of the Acacia honey, diastase activity of the Acacia, Ziziphus and polyfloral honey and invertase activity of the polyfloral honey (Table1).

**Table 1:** The results of the studied parameters in the investigated honey samples and the geographical effect on them.

Parameters of honey	Types of honey	N	Mean	Std. deviation	Minimum	Maximum	p-value
Moisture %	Acacia Asir	4	17.64	2.04	14.80	19.25	0.02
	Acacia Jazan	4	14.70	0.39	14.25	15.10	
	Ziziphus Asir	4	17.25	1.85	14.50	18.5	0.85
	Ziziphus Jazan	3	17.50	0.78	17.00	18.40	
	Polyfloral Asir	12	17.68	1.50	15.40	19.60	0.47
	polyfloral Jazan	14	17.18	1.99	14.20	19.50	
	Total	41	17.16	1.80	14.20	19.60	
Conductivity mS/cm	Acacia Asir	4	0.80	0.397	0.560	1.390	0.13
	Acacia Jazan	4	0.45	0.229	0.130	0.630	
	Ziziphus Asir	4	0.65	0.413	0.035	0.910	0.63
	Ziziphus Jazan	3	0.53	0.240	0.380	0.810	
	Polyfloral Asir	12	0.77	0.424	0.210	1.420	0.03
	polyfloral Jazan	14	0.49	0.142	0.160	0.720	
	Total	41	0.62	0.327	0.035	1.420	
Acidity meq of NaOH/Kg	Acacia Asir	4	18.75	4.79	15.00	25.00	0.88
	Acacia Jazan	4	17.50	2.89	15.00	20.00	
	Ziziphus Asir	4	26.25	16.52	15.00	50.00	0.47
	Ziziphus Jazan	3	20.00	13.23	10.00	35.00	
	Polyfloral Asir	12	22.50	6.91	10.00	30.00	0.08
	Polyfloral Jazan	14	30.34	13.93	10.00	50.00	
	Total	41	24.51	11.44	10.00	50.00	
Fructose + glucose %	Acacia Asir	4	70.52	2.79	68.26	74.57	0.06
	Acacia Jazan	4	62.86	3.35	60.26	67.37	
	Ziziphus Asir	4	74.86	6.06	70.86	83.88	0.08
	Ziziphus Jazan	3	66.92	8.96	61.03	77.23	
	Polyfloral Asir	12	73.22	7.20	60.53	84.07	0.35
	Polyfloral Jazan	14	71.12	4.19	65.25	78.64	
	Total	41	70.93	6.25	60.25	84.07	
Sucrose %	Acacia Asir	4	4.68	3.21	0.00	6.80	0.02
	Acacia Jazan	4	1.82	0.28	1.41	2.01	
	Ziziphus Asir	4	3.37	2.94	0.54	6.20	0.29
	Ziziphus Jazan	3	2.03	0.30	1.72	2.32	
	Polyfloral Asir	12	1.71	1.56	0.00	3.94	0.20
	Polyfloral Jazan	14	2.56	0.85	0.00	3.24	
	Total	41	2.49	1.77	0.00	6.80	
Diastase DN	Acacia Asir	4	21.44	14.22	10.87	42.43	0.001
	Acacia Jazan	4	4.99	2.75	3.01	9.05	
	Ziziphus Asir	4	12.27	7.02	5.65	21.31	0.046
	Ziziphus Jazan	3	2.44	1.08	1.20	3.15	
	Polyfloral Asir	12	16.17	6.48	4.81	26.56	≤ 0.001
	Polyfloral Jazan	14	2.90	2.96	0.94	12.50	
	Total	41	9.68	9.12	0.94	42.43	
pH	Acacia Asir	4	5.25	0.33	5.00	5.70	0.21
	Acacia Jazan	4	4.60	0.27	4.20	4.80	
	Ziziphus Asir	4	4.78	0.82	3.80	5.50	0.45
	Ziziphus Jazan	3	4.60	0.27	4.20	4.80	
	Polyfloral Asir	12	4.53	0.76	3.70	6.00	0.06
	Polyfloral Jazan	14	3.96	0.49	3.60	4.70	
	Total	41	4.49	0.81	3.60	7.20	
Fructose %	Acacia Asir	4	44.76	1.64	43.27	46.44	0.01
	Acacia Jazan	4	36.50	1.43	34.61	37.62	
	Ziziphus Asir	4	41.64	4.19	35.30	44.81	0.86
	Ziziphus Jazan	3	40.53	9.09	34.51	50.98	
	Polyfloral Asir	12	42.99	3.76	38.74	49.85	0.68
	Polyfloral Jazan	14	42.26	5.00	35.39	49.86	
	Total	41	41.92	4.71	34.51	50.98	
Glucose %	Acacia Asir	4	26.76	1.78	24.04	18.13	0.84
	Acacia Jazan	4	26.37	4.78	22.66	32.76	
	Ziziphus Asir	4	33.72	5.52	27.76	40.46	0.03
	Ziziphus Jazan	3	26.39	1.54	24.93	27.99	
	Polyfloral Asir	12	30.22	5.55	19.19	38.89	0.40
	Polyfloral Jazan	14	28.86	2.37	25.54	32.29	
	Total	41	29.01	4.42	19.19	40.46	
Fructose/Glucose ratio	Acacia Asir	4	1.56	0.32	1.20	2.34	0.70
	Acacia Jazan	4	1.66	0.27	1.27	1.87	
	Ziziphus Asir	4	2.22	0.47	1.90	2.90	0.035
	Ziziphus Jazan	3	1.37	0.23	1.20	1.53	
	Polyfloral Asir	12	1.67	0.80	1.07	3.54	0.38
	Polyfloral Jazan	14	1.49	0.28	1.19	1.94	
	Total	41	1.62	0.47	1.07	3.54	
Invertase IN	Acacia Asir	4	59.21	69.05	5.30	152.74	0.31
	Acacia Jazan	4	20.58	8.54	8.26	26.86	
	Ziziphus Asir	4	113.72	96.88	5.56	209.64	0.11
	Ziziphus Jazan	3	46.52	40.40	1.75	80.26	
	Polyfloral Asir	12	119.44	67.69	6.43	236.83	≤ 0.001
	Polyfloral Jazan	14	13.48	9.89	2.54	29.24	
	Total	41	61.84	68.64	1.75	236.83	

### Effect of the floral origin on the studied parameters of the honey samples

**Figure 2:**
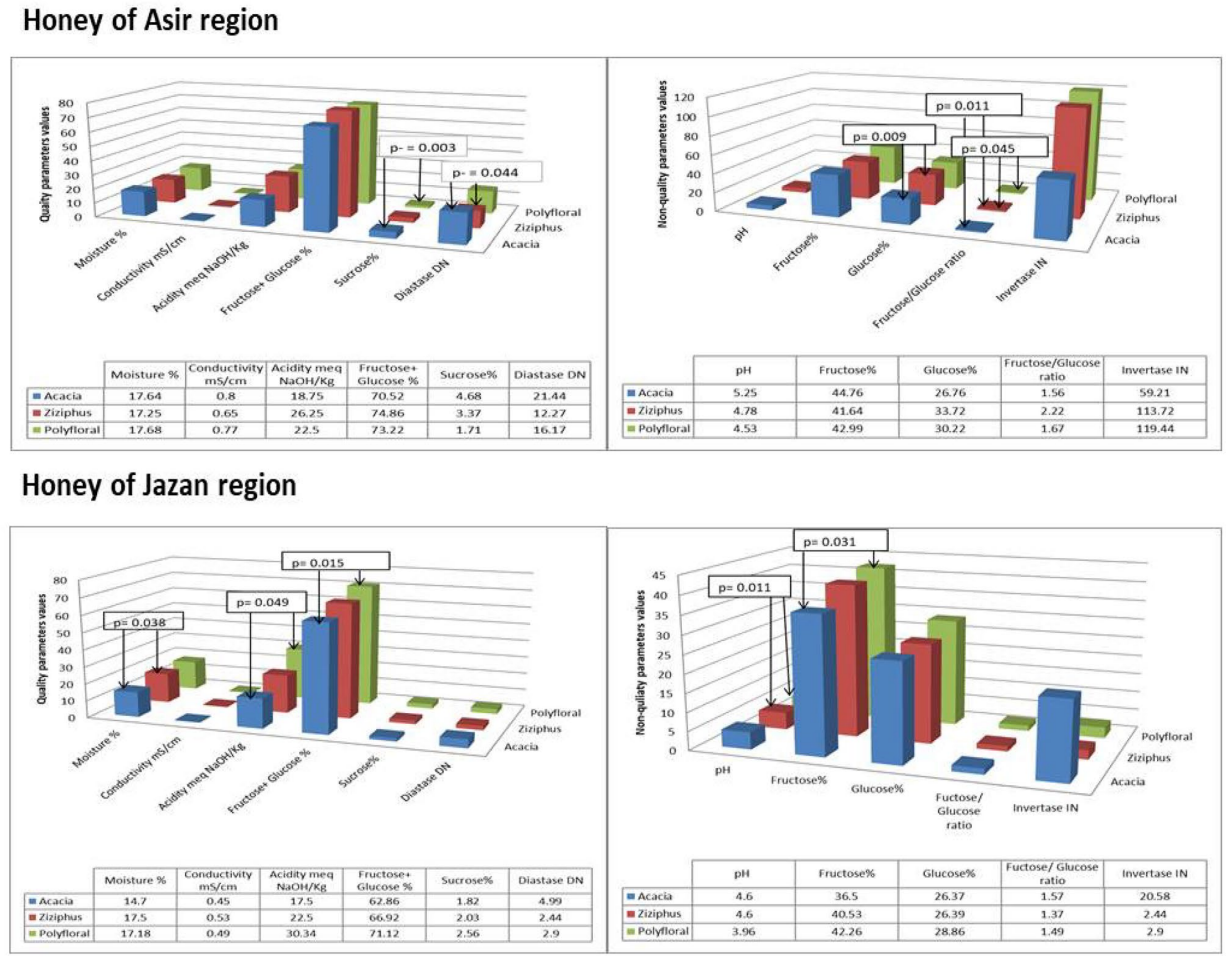
Effect of the floral origin in Asir and Jazan region on the studied parameters of honey.

The floral origin had more significant effects on the quality parameters of the honey samples from Jazan region compared to the honeys of Asir region. Concerning the quality parameters of the honey samples from Asir region, the sucrose percentage was significantly increased in the Acacia honey compared to the polyfloral honey (p-value = 0.003), the diastase activity of the Acacia honey was significantly increased compared to the Ziziphus honey (p-value = 0.044). With regard to the non-quality parameters of Asir honey, the glucose percentage was significantly decreased in the Acacia honey compared to the Ziziphus honey (p-value = 0.009) and the fructose/glucose ratio of the Ziziphus honey was significantly more than that of the Acacia honey (p-value = 0.011) and ployfloral honey (p-value = 0.045) (Fig. 2). The mean values of the studied quality parameters of the honey samples from Jazan region showed that the moisture percentage was significantly increased in the Ziziphus honey compared to the Acacia honey (p-value = 0.038), the acidity of the Acacia honey was significantly decreased compared to that of polyfloral honey (p-value = 0.049) and the sum of fructose and glucose percentages of the Acacia honey was significantly decreased compared to the polyfloral honey (p-value = 0.015). The significantly affected non-quality parameters of Jazan honey were the pH of the Ziziphus honey compared to that of the polyfloral honey (p-value = 0.011) and the fructose percentage of the Acacia honey if compared to the polyfloral honeys (p-value = 0.031) (Fig. 2).

### Agglomerative hierarchical clustering analysis results

Generally, there are two types of data clustering (K-means); centroid based clustering which cluster data in a centric pattern such as the density based clustering and distribution based clustering and hierarchical or tree clustering^21^. Hierarchical clustering is classified to agglomerative and divisive clustering. Hierarchical agglomerative analysis classifies data from the bottom (each datum has its own singleton cluster), merges singleton clusters to form group clusters, merges groups into upper level groups and finally, merges the upper level groups to involve all the study community. Divisive clustering starts with the whole data and starts dividing the whole data group into subgroup until it reaches the singleton cluster groups^22^.

**Figure 3:**
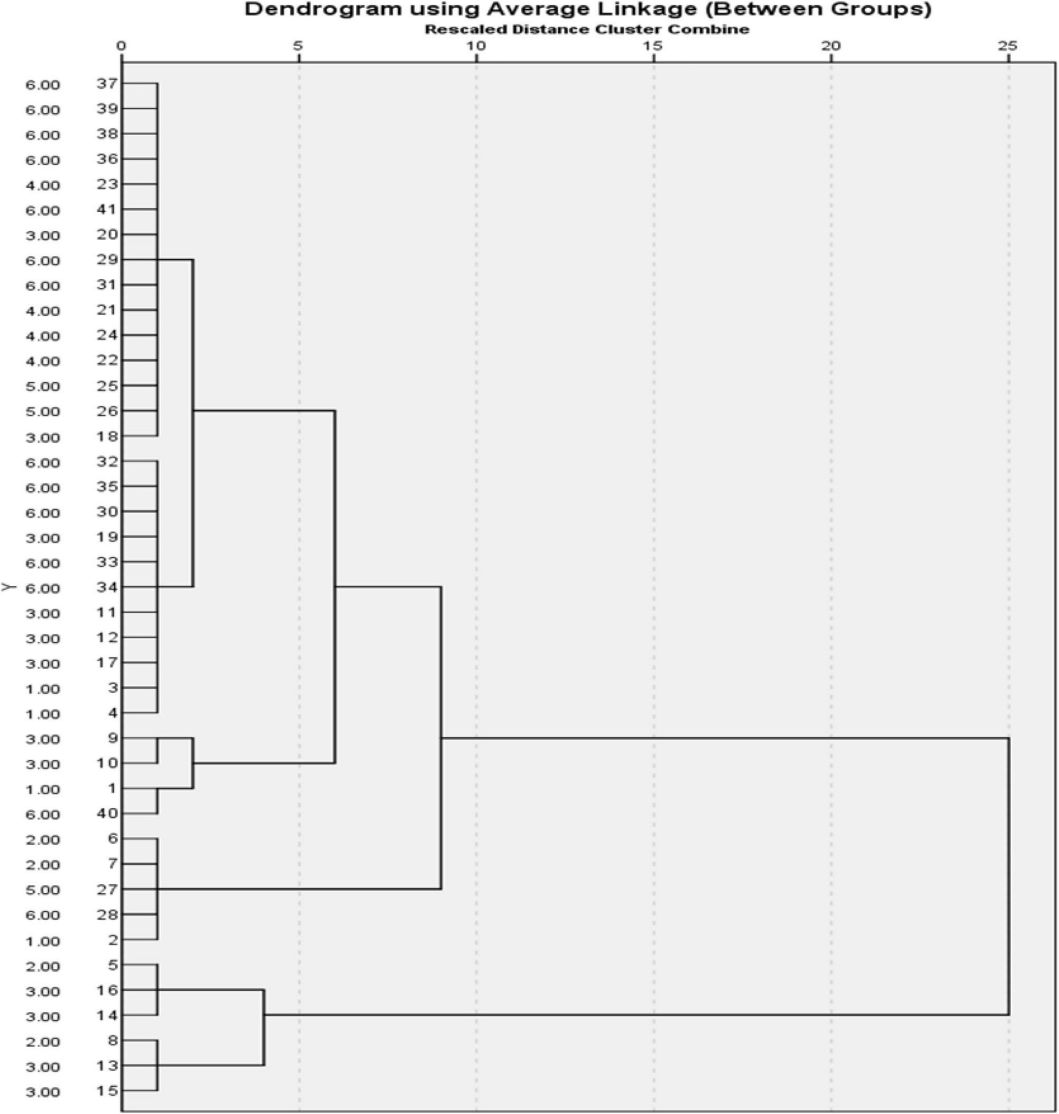
The Agglomerative hierarchical clustering results. This tree clustering analysis was carried out to examine the efficiency of the studied honey quality parameters in predicting the floral and geographical origin of the honey samples (100%).

The agglomerative hierarchical clustering was effective in clustering all the singleton clusters into the whole data cluster (100%) (Fig. 3). There were three groups of singleton clusters from up to down. Group one contained 26 honey samples (19; 73.08% from Jazan and 7; 26.92% from Asir). Floral wise, group one contained 18 (69.2%) polyfloral honey samples (12 from Jazan, 6 from Asir), 6 (23.1%) Acacia honey samples (4 from Jazan and 2 from Asir) and 2 (7.7%) Ziziphus honey samples from Jazan. Group 2 contained three honey samples from Asir region (100%). Two of group 2 were polyfloral honey samples and one was Acacia honey. Group 3 was composed of six honey samples from Asir region (100%). The floral origins of group three were polyfloral (4; 66.7%) and ziziphus (2; 33.3%) (Fig. 3).

The second level of clustering contained one group that clustered group one and two from the first level (29 honey samples; 70.7%). Geographical wise, this group contained 18 samples from Jazan (62.1%) and 11 samples from Asir region (37.9%). The floral origins of the samples from Jazan were Acacia (4), Ziziphus (2) and polyfloral (12). The floral origins of Asir honey samples were Acacia (3), Ziziphus (0) and polyfloral (8) (Fig. 3).

The third level of clustering was composed of one group involving the second level group and six singlet clusters; Three from Asir (2 Ziziphus and one Acacia) and three from Jazan (one Ziziphus and two polyforal). The total number of samples in the third level group was 34 (82.9%). The third level group comprised 20 samples from Jazan (58.8%) and 14 samples from Asir (41.2%). The floral origins of Jazan honey samples were Acacia (4), Ziziphus (3) and polyfloral (14) while those of Asir region were Acacia (4), Zizphus (2) and polyfloral (8) (Fig. 3).

The fourth level of clustering involved the third level group and group 3 of the first clustering level. This cluster group involved all the studied honey samples 21 from Jazan (4 Acacia, 3 Ziziphus and 14 polyfloral) and 20 from Asir (4 Acacia, 4 Ziziphus and 12 polyfloral) (Fig. 3).

### Multiple linear regression analysis results

**Figure 4:**
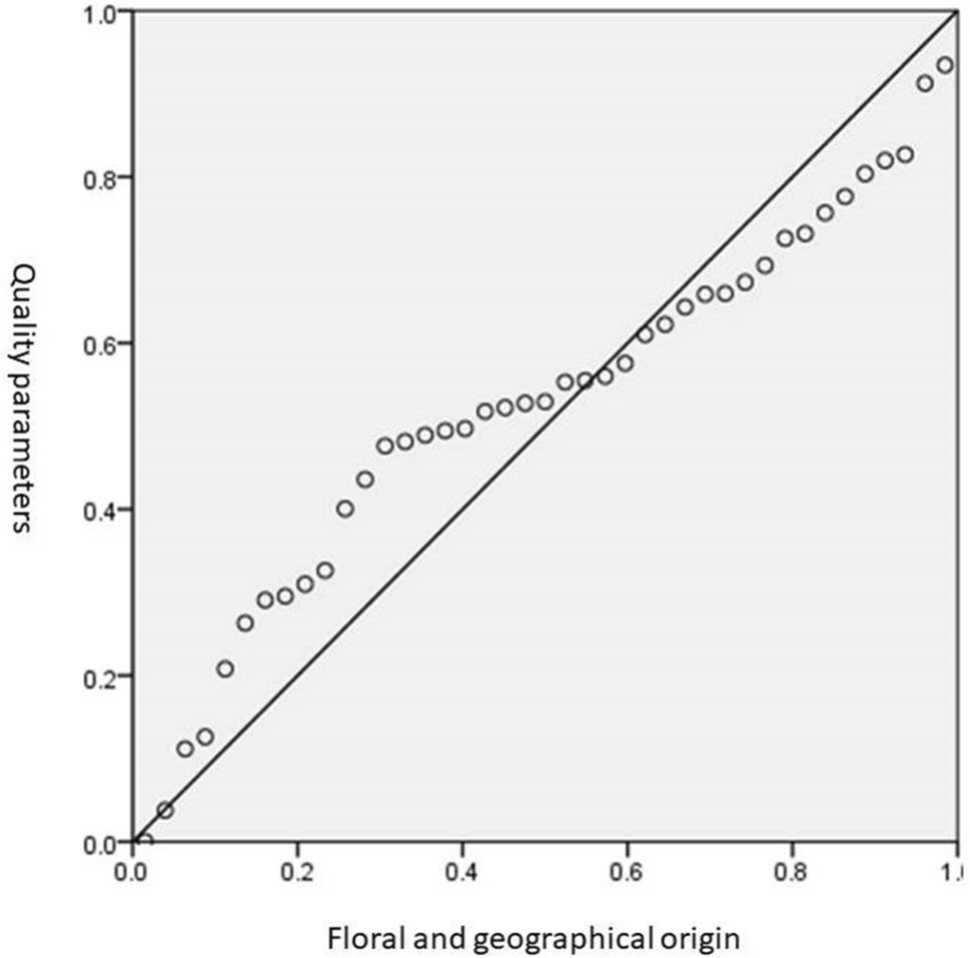
Multiple linear regression analysis result. The honey quality parameters significantly predicted the floral and geographical origin of the honey samples (F (9, 31) = 7.031, p < 0.0001, R2 = 0.671, R = 0.819).

The results of the honey moisture, conductivity, pH, acidity, fructose, glucose, sum of fructose and glucose, sucrose, diastase and invertase significantly predicted the floral and geographical origins of the honey samples, F (9, 31) = 7.031, p < 0.0001, R^2^ = 0.671. All ten variables added statistically significantly to the prediction (Fig. 4). Moreover, The floral and geographical origin of the honey samples is directly correlated to the results of the ten parameters (R = 0.819).

## Discussion

This study reported that the studied honey samples were not adulterated since their quality parameters comply with the international and national honey standards as follows: (1) the moisture of the honey samples of this study was less than 20% which comply with international, regional and local Saudi honey standards^1,4^; (2) the range of the electrical conductivity of the honey samples was within the ranges of the international and local standards (35.0–1420 S/cm) ^1,4^; (3) the free acidity range of the honey samples was (10.0–50.0 meq of NaOH/Kg) complying with the ranges in the honey standards^1,3,4^; (4) the sum of fructose and glucose is included as a honey quality parameter in all of the honey standards with the value of (≥ 60%) in nectar honey and (≤ 45%) in the honeydew honey^1,3,4^. The range of the total fructose and glucose in this study was (60.25– 84.07%) which is comparable to the honey standards and comparable to the results of previous studies (39.6–78.13%), (50.26–74.74%), (62.24–79.92%), (82.07–84.35%) and (63.4–94.1%) ^23–27^; (5) the range of the sucrose percentage in the honey samples of this study was (≤ 6.80%) which falls within the ranges of the honey standards^1,3,4^; (6) the diastase activity of the studied honeys was ranging from 0.94 to 42.43 DN. The mean values Diastase of Ziziphus (2.44 ± 1.08 DN) and polyfloral (2.90 ± 2.96 DN) honeys from Jazan region were less than 3 DN which does not comply with the standards. The low diastase activity of the Jazan honey samples may be due to the hot climate of Jazan, the floral origin or presence of indigenous molecules that inhibit the diastase ^10,28,29^. Furthermore, some studies reported very low activity of diatase in honey samples ranging from 0.00 to 2.5 which is far less than the standard activities of diastase in honey^30–32^. The university of Waikato published a Master thesis in which it concluded that manuka honey is characterized by low activities of diastase which may be due to presence of methylglyoxal or phenyllactic acid and suggested that the honey regulations may be not proper for the evaluation of Manuka honey^33,34^. The honey standards are better to reinvestigate the diastase activity in honey samples from hot climate areas. (7) The range of the pH of the studied samples was (3.6–7.2). however, the pH is not considered as a honey quality parameter in all the honey standards except the Honey reference guide issued by the national honey board- USA which reported that the pH of the honey range is (3.4–6.1)^1,3,4,35^. Some published article reported several pH values of honey such as 6.5, 6.8, 6.89, 6.97, 7.2 and 7.4^36–38^; (8) with regard to the fructose and glucose percentages, they are not included as a quality parameters of honey authentication. The fructose and glucose ranges were (34.51–50.98%) and (19.19–40.46%), respetively. Ghramh^39^ found that the fructose concentration in honey samples from Asir region was (33.10–44.77%). The upper level of fructose percentage in our samples was higher than that reported by Ghramh and his research group which may be due to the increased number of samples in our study compared to their study and due to inclusion of honey samples from Jazan region. Similarly, Aljohar^23^, Alghamdi^24^ and Baloš^25^ reported a fructose range of (2.636–39.143%), (29.08–40.63%) and (36.57–41.64%) in Saudi and Serbian honey samples, respectively. The range of the glucose percentage in this study was (19.19–40.46%) was comparable to the ranges of the previous studies of Ghramh^39^ (26.68–37.91%), Aljohar^23^ (16.265–42.84%), Alghamdi^24^ (20.02–34.1%) and Baloš^25^ (30.00–34.53%); (9) the ratio of fructose/glucose is used to predict the crystallization tendency of honey samples. This study reported a range of fructose/glucose ratio of (1.07–3.54) with the mean value of 1.62 ± 0.47. Our result is within the range stated by the review article of Bobiş et al.^9^. However, the upper limit of this study is too high (3.54) which may be due to the activity of the glucose metabolizing enzymes such as the glucose oxidase and (10) invertase activity is not considered as a honey quality parameter in the international and regional standards. It is adopted as a quality parameter in some European countries as indicator of honey freshness with the range of ((≥ 10 IN)^16^.

With regard to the effect of the geographical origin on the studied quality parameters, it had significant effects as follows: (1) this study reported that the Acacia honey of Jazan was characterized by significantly decreased moisture percentage compared to the moisture percentage of the Acacia honey from Asir (Table1). Honeys of hot climate are expected to be with low moisture percentage since its treatment with temperature is reported to decrease its moisture percentage^40^. Many studies proved that the geographical origins has strong effect on the physicochemical properties of honey including the moisture percentage^20,41–43^. (2) The geographical origin significantly affected the conductivity of the polyfloral honey (0.77 mS/cm for Polyfloral Asir and 0.49 mS/cm for Polyfloral Jazan) (p-value = 0.03) (Table1). Acquarone^43^ and Adgaba^45^ stated that the conductivity of honey samples is useful in predicting their geographical origin. (3) The geographical origin had insignificant effect on the acidity of the honey samples of this study. Similarly, Tomczyk et al. ^46^ reported that honey samples of the same floral origins from Poland and Slovakia were significantly different in their acidity concentration. (4) In this study, the geographical origin did not exerted significant effect on the sum of fructose and glucose. Unlike our conclusion regarding the geographical effect on the total of fructose and glucose, Scholz^42^ and Agussalim^47^ concluded that the monosaccharides are useful in the differentiation between honey samples of different geographical origins. (5) The mean concentration of the sucrose of the Acacia honey from Asir region (4.68%) was significantly more than that of Acacia Jazan (1.82%) (p-value = 0.02). The result shows the significant effect of the geographical origin on the sucrose percentage (Table1). Similar to our finding, Karabagias^48^ reported significant differences in the sucrose percentages of pine and fir honeys from different geographical origins. (6) The geographical origin significantly affected the diastase activity of the Acacia, Ziziphus and polyflral honeys (p-value = 0.001, p-value = 0.046 and p-value ≤ 0.001, respectively) (Table 1). The diastase activity of honey samples of the same floral origin and different geographical origins was reported to be significantly different^46^. (7) This study found that the geographical origin had insignificant effect on the pH of the honey samples (the pH cannot predict the geographical origin). Some previous studies showed that the pH differs significantly and may be used to predict the geographical origin of honeys^41–43^. (8) The fructose percentage in the Acacia honey from Asir region was significantly more than that of the Acacia honey from Jazan region (p-value = 0.01). Similar to our finding, it was reported that the geographical origin of honey has significant effect on its fructose percentage^41–46^. (9) The glucose percentage of the Ziziphus honey from Asir region (33.72%) was significantly increased compared to its percentage in the Ziziphus honey from Jazan region (26.39%) (Table 1). Monosaccharide sugars are well known to be useful markers for the prediction of the geographical origin of honey^42,45^. (10) The geographical origin significantly affected the fructose/glucose ratio of the Ziziphus honey (p-value = 0.035) (Table 1). The significant effect of the geographical origin on the fructose/glucose ratio was previously reported by Agussalim^45^. (11) The polyfloral honey of Asir region was characterized by increased invertase activity (119.44 IN) compared to that of the polyfloral honey of Jazan region (13.48 IN) (p-value ≤ 0.001) (Table 1). Similar to the finding of this study, Boussaid^47^ stated that the geographical origin of honey has significant effect on the invertase activity.

The effect of the floral origin on the studied parameters was as follows: (1) the moisture percentage of the Acacia (14.7) and Ziziphus (17.5) of Jazan were with significantly different moisture content. Many of the previous studies showed that the moisture percentage of honey depends on its floral origin^26,38^. (2) The floral origin has insignificant effect on the honey conductivity in Asir and Jazan (Fig. 2). Majewska^48^ concluded that the conductivity and ash of honey are best predictors for linden and buckwheat honeys but not Acacia and Rapeseed honeys. (3) The acidity of the Acacia honey from Jazan was significantly less than that of the Jazan polyfloral honey [Fig. 2]. Similar to our conclusion, Acquarone^42^ concluded that the honey acidity and ash are useful in the differentiation between honeys of different floral origin in a given geographical origin. Majewska^48^ did not find that the honey acidity is useful in discriminating between the different floral origins of honey. (4) The total of fructose and glucose in the Acacia honey from Jazan was 62.86% compared 71.12% in the polyflroal honey from Jazan with significant variation (p-value = 0.015). Escuredo^49^ concluded that the total of fructose and glucose is a predictor of the floral origin of honey samples. (5) Regarding the floral origin effect on the sucrose percentage, it had no significant effect either in Asir or Jazan regions. Schiassi et al.^50^ reported significant variation in sucrose percentage between honey samples of different floral origins. (6) The diastase activity of the Acacia honey from Asir region was significantly increased compared to the Ziziphus honey of the same region (p-value = 0.044) (Fig. 2). Meskele^30^ and Tomczyk^44^ concluded that the diastase activity is significantly different between honeys of different floral origins within the same geographical origin. (7) The floral origin had significant effect on the pH of the honey samples. The Ziziphus honey of Jazan region was characterized by increased pH value compared to the Jazan polyflroal honey (p-value = 0.011) (Fig. 2). Unlike our finding, Acquarone^42^ and Majewska^48^ reported that the pH is not useful in predicting the floral and geographical origin of honey samples. (8) The Acacia honey from Jazan was characterized by significantly decreased fructose percentage (36.5%) compared to the fructose percentage of the Jazan polfloral honey (42.26%) (p-value = 0.031) (Fig. 2). Floral origin of honey is known to be predicted its monosaccharide and oligosaccharide concentration including the fructose^51^. (9) We did not report significant effect of the floral origin on the glucose percentage of honey. However, Tedesco^51^ stated that mono and oligosaccharides discriminate between the floral origins of honey samples. (10) The ratio of fructose/glucose of the Asir Ziziphus honey was significantly more than that of the Acacia and polyfloral honeys from Asir region (p-values = 0.011 and 0.045, respectively) (Fig. 2). The fructose/glucose ratio is reported to differentiate between honey samples from different floral origns^25,49,52^. (11) The floral origin had insignificant effect on the invertase activity of the honey samples from Asir and Jazan regions (Fig. 2). Unlike our conclusion that the floral origin has insignificant effect on the invertase activity, Makhloufi^53^ conveyed that the floral origin has significant effect on the invertase and diastase activities.

The effect of the floral origin on the honey quality and non-quality parameters is more in the honeys from Jazan region than those from Asir region. The difference in the effects may be due to the effect of the hot climate characteristics of Jazan and the cold climate of Asir region. We did not find one study combining the effect of floral origin on the quality parameters of honey samples from hot and cold climate regions. Instate, we found researches that studied the effect of the floral origin in hot climate region alone or cold climate regions alone. In subtropical and mesothermic region the floral origin is proved to significantly affect the pH, acidity, electrical conductivity, diastase activity, sucrose percentage and reducing sugars^41^. In equatorial hot climate, the floral origin has significant effect on sugars, ash and moisture^54^. In the high altitude region of Himalaya, honey samples of multifloral origins are reported to be different regarding their moisture percentage, sucrose, ash and free acidity^55^.

Concerning the result of the Hierarchical clustering analysis, our study proved the effectiveness of the honey quality parameters in predicting the geographical and floral origins of the honey samples. Many previous studies reached to the same conclusion by applying different chemometric and clustering analysis tests such as the principal component analysis (PCA) and the partial least square discriminant analysis (PLS-DA)^27,38,39,41,43,44,48^.

In this study, we applied multiple linear regression analysis to examine the possibility of using the quality parameters results to predict the geographical and floral origins of the honey samples. The multiple linear regression analysis was very effective in examining the usefulness of the quality parameters in identifying the geographical and floral origins collectively.

This study is associated mainly with the honey standards and the authorities responsible for the derivation of them. The available honey standards concentrate on the floral origin of the honey samples to set the standards except or the hydoxymethylfurfural (HMF) which is set according to the floral and climate conditions^1,3,4^. Regarding the HMF level in the honey, the honey standards set two levels; one for tropical regions and one for the non-tropical regions. The HMF of honey from tropical regions should not exceed 80 mg/Kg while for non-tropical regions should not exceed 40 mg/Kg^1,3,4^. This article calls for the honey standards authorities to consider both the floral and climate origins in the future versions of the standards. Hot climates are known to impact the physicochemical properties and chemical composition of honey depending on the floral origin since some unifloral honeys are resistant to high temperatures while others are sensitive to temperature change^56,57^. Furthermore, the effect of honey biomolecules on each other’s is proved by many studies such as the manuka honey which is known to be rich in methylglyoxal that is capable of inhibiting some enzymes such as the diastase and catalase^20,34,58^. Considering the revision of the honey standards through combining of both the floral and geographical origins may help the beekeepers and honey producers in the marketing of their honey.

The suggested revision for the honey standards is expected to reach the same conclusion of the previous studies which will lead to changing the concentration of the standards. As example, we expect to minimize the diastase activity according to the geographical origin and honey composition. Moreover, this study suggests including other parameters for honey authentication such as the mineral content, total phenolic acids, amino acids, flavonoids and antioxidant activities. These parameters are reported by many studies to be useful in predicting the floral and geographical origins of honey^26,28,43,44,59–63^. However, it is important to include new honey standards after extensive laboratory analysis for honey samples from variable floral and geographical origins covering all the beekeeping areas in the globe.

## Conclusion

The geographical and floral origins jointly have variable significant effects on the studied quality and non-quality parameters of honey. The joint effect of the geographical and floral origins on the quality parameters of honey is majorly due to the climate condition of the geographical origin. Hot climate conditions affect the enzyme calalyzed and non-enzymatic reactions in honey which is known to impact the physicochemical characteristics and the quality parameters of the honey. Moreover, some unifloral honeys are known to resist changes in climate and storage conditions while others are vulnerable. The scope of the floral origin and geographical origin effects are regulated by each other. Honey samples from Jazan region had low diastase activity which may be due to the hot climate or presence of chemicals that may inhibit the diastase. Lowering the diastase activity in the standards for the honey samples from hot climates such as Jazan region will help the beekeepers and honey producers in marketing their honey locally and abroad. We suggest for the international honey commission to consider revising the honey standards by joining the effects of floral and geographical (climate zones) origins and move from depending on the floral origin alone to derive honey standards. Our suggestion depends on three reasons: (1) in the late version of the Codex honey standards, the HMF concentration was set in two values according to the climate conditions, (2) published articles regarding the diastase activity in manuka honey and (3) the findings of this study.

## Methods

### Study area

Asir region is located in the southwestern Saudi Arabia at 41–45° E longitude and 17–21° N latitude. It is found on a highland that contains the Saudi’s highest mountains. The altitude of Asir region rises from the sea level up to 3000 m at Al-souda Mountains. The mountains of Asir region has a foggy climate which leads to the dominance of coniferous trees and dense forests. The temperature in this region is low (12–35 °C) and the rainfall is more (300 mm mean amount annually) compared to the other regions of Saudi Arabia^64,65^.

Jazan is a southwestern region of Saudi Arabia located 42° 33′ 4′′ E longitude and 16° 53′ 21′′ N latitude. It is bordered by Asir region on the North and the Red Sea on the West. The Jazan region is the richest agricultural part of Saudi Arabia because it contains different environments such as the agricultural lands, mountains, valleys, deserts and semi deserts. It has a 300 km red sea coast and over 100 islands. Jazan region is well known by its hot desert climate, high humidity and high barometric pressure with an average temperature of 30 °C and a rainfall range between 70 and 270 mm annually^56,66,67^.

### Study design and honey samples

This study can be classified as descriptive, quantitative and case control study design. Descriptive studies are associated with the description of distribution of one or more variables without investigating to the causal effects. There are different types of descriptive studies including case reports, cross-sectional studies and ecological or correlational studies^68^. This study is considered quantitative since it involves quantitative experiments and it is case control because it compares between samples of different geographical and floral origins^69^. Forty one honey samples were collected for Asir and Jazan regions directly from their bee farms and in their hives to avoid adulteration. Moreover, the floral origin of the honey samples was confirmed microscopically through pollen analysis according to Louveaux^70^. A honey sample is considered monofloral if one pollen type constitute more than 50% of the whole pollens. The honey samples from Asir region were Acacia (4), Ziziphus (4) and polyfloral (12) while those of Jazan region were 4, 3 and 14, respectively. Microscopic confirmation of the floral origin of the honey samples through determining the dominant pollens is a very important step in this research because the floral origin is the major factor that determines the honey quality, physicochemical properties and sensory characteristics^1,3,4,71^.

### Analysis procedures of the studied quality parameters

The studied quality parameters of honey were analyzed according to the harmonized methods of international honey commission^72^ while the sugar profile was determined following the instructions of Agilent technologies HPLC method^53^.

### Moisture percentage

The moisture percentage of the honey samples was determined according to the refractometric method of the harmonized methods of international honey commission^73^.

### Electrical Conductivity (EC)

The EC (µS/cm) was measured following the steps of the harmonized methods of the international honey commission^74^.

### pH and free acidity

To prepare the honey solution for the measurement of the free acidity, two grams of each honey sample were dissolved in 15 mL distilled water to reach the dilution of 13.3% (w/v).

The solution was titrated with standardized 0.1 M NaOH to a final pH of 8.3. The concentration of acids in honey was expressed in meq of acid per kg of honey using the bellow equation^75^. The method was slightly modified compared to the original method through taking two grams of honey instead of 10 g and accordingly the equation is changed.

$$ {\text{acidity}}\left( {\frac{meg}{{kg}}} \right) = \frac{volume \;of\; 0.1M NaOH \;consumed*50 }{{kg \;of \;honey \;samples}} $$

### Sugar profile

The main sugar composition (fructose, glucose, sucrose) in honey was determined following the method of Agilent technologies using the HPLC (Agilent 1260 Infinity II, Agilent Technologies, California, USA) equipped with a detector (refractive index detector (1260 RID, Agilent Technologies, California, USA) at 35 °C. Honey sample (1.25 g) was dissolved in 25 mL HPLC grade water, mixed with magnetic stirrer then filtered through a 0.22 micron filter and injected (10 μL) into the HPLC system. The HPLC column (ZORBAX Carbohydrates: (4.6 × 150 mm; 5 μm) Agilent Technologies, California, USA), the mobile phase was acetonitrile: water (75:25, v/v), at a flow rate of 1.0 mL/min and spray volume of 10 µL. Standard solutions of fructose (2%; W/V), glucose (2%; W/V) and sucrose (1%; W/V) were used as standards to help calculating their concentration in the honey samples^76^.

### Determination of diastase activity

#### The diastase assay principle and reagents

The principle of this test is that the diastase of the honey reacts with starch solution and converts it to glucose and maltose. Iodine reagent is added to forms a blue color with starch. Sodium chloride is added so as to increase the diastase activity and acetate buffer is used to adjust the pH of the reaction^77^. The concentration of the starch decreases according to the activity of the honey diastase. The intensity decrease of the blue color is recorded in constant intervals and a plot is created between the absorbance and time. Tx is the time for the diastase to reach the absorbance value of 0.235. The diastase number (DN) is determined by dividing 300 by the Tx value^77^. The reagents of the experiment were prepared according to the instructions of the International honey commission methods of honey analysis (Table 2).

**Table 2:** Reagents of the measurement of the diastase activity.

Reagent	Preparation method
Starch (Substrate for the diastase)	(1) 2 g of starch were weighted and heated at a temperature 130 °C for 90 min (2) 90 mL of distilled water were added and mixed well by stirring (3) The above solution was quickly transferred to 100 mL volumetric flask and placed on a hot water bath and boiled for three minutes gently with constant stirring (4) The boiling solution was cooled by placing the volumetric flask in running water at room temperature and the volume was made up to 100 mL with distilled water **Note:** a fresh starch solution was prepared every day because the starch solution retrogrades overtime and microorganisms grow in it
Sodium chloride (Diastase activity enhancer)	2.9 g of NaCl were dissolved in distilled water and diluted to 100 mL using a 100 mL volumetric flask
Acetate buffer (To adjust the pH)	43.5 g of sodium acetate (NaCH2COO.3H2O) were dissolved in distilled water and the pH was adjusted to 5.3 by adding about 5 ml of glacial acetic acid. The solution was transferred to a 250 mL volumetric flask and diluted with distilled water
Iodine stock	11 g of iodine and 22 g of potassium iodide were dissolved in 30 to 40 ml of distilled water, diluted to 500 ml in a volumetric flask and stored in dark bottle
Iodine solution (chromogenic)	20 g of potassium iodide were dissolved in distilled water, 2 ml of iodine stock solution were added and the volume was made up to 500 ml
Sample preparation	10 g of honey were dissolved in 15 ml of distilled water, 5 ml of acetate buffer were added. The mixture was transferred to a 50 ml volumetric flask containing 3 ml of NaCl solution. Finally, the volume was adjusted 50 mL with distilled water

#### The diastase assay procedure

The first step is to determine the amount of water to be added to the prepared sample (starch titration). A series of beakers containing 20, 21, 22, 23, 24 and 25 mL of water were prepared and 5 mL were added from a mixture containing 10 mL distilled water and 5 mL starch solution. After that 5 mL of iodine solution were added and the absorbance was read. The water volume which read in the range of (0.745–0.770) at the wavelength of 660 nm was chosen for the dilution of the prepared honey samples^77^.

Secondly, 10 mL of honey solution and 10 mL of starch solution were taken in separate beakers and placed in water bath at 40 °C for 15 min. 5 mL from the starch solution were added to the 10 mL of honey solution and mixed thoroughly. After 5 min, 500 µL from the sample/starch solution were transferred into a beaker containing 5 mL of iodine solution plus the amount of distilled water selected according to the starch titration. The absorption of the above solution (sample/starch/iodine) was immediately read at 660 nm against distilled water as a blank^77^.

Thirdly, the second step was repeated every 5 min until the absorption was less than 0.235 by three readings. A plot was created between the time and the absorbance and straight line equation was derived. From the line equation the time (Tx) needed to obtain the 0.235 absorbance was determined. The diastase number (DN) was calculated by dividing the number 300 by the Tx value^77^.

$$ DN = \frac{300}{{{\text{Tx}}}} $$

DN = Diastasis Number. Tx = The time in minutes needed to reach the 0.235 absorbance.

### Determination of invertase activity

#### The invertase assay principle and reagents

The harmonized method of international honey commission was followed to determine the invertase activity^78^. The chemical compound p-Nitrophenyl-a-d-glycopyranoside (pNPG) was used as a substrate to measure the activity of the invertase. The invertase catalyzes the hydrolysis of the substrate (pNPG) and converts it to glucose and nitrophenol. The reaction was stopped by adjusting the pH to 9.5 via a reaction-terminating solution which inhibits the activity of the enzyme while converting the nitrophenol compound to the nitrophenol anion. The nitrophenol anion concentration was measured spectrophotmetrically at 400 nm^78^.

The reagents of the invertase assay include: (1) phosphate buffer (0.1 M, pH 6) (11.66 g of potassium hydrogen phosphate KH2PO4 and 2.56 g of disodium hydrogen phosphate Na2HPO4∙2H2O were dissolved in distilled water and diluted to 1L); (2) substrate p-nitrophenyl-α-d-glucopyranoside (pNPG) (0.02 M) (6.0252 g of pNPG were liquefied in buffer solution, heated not above 60 °C, cooled, diluted to one liter and cooled immediately. The solution was stored in the refrigerator for one month) and 3) Reaction-terminating (3 M, pH 9.5) (363.42 g of tris- (hydroxymethyl) aminomethane were dissolved in distilled water, diluted to one liter and the pH was adjusted to 9.5 using 3 M HCL)^78^.

To prepare the honey samples for the measurement of invertase activity, 5 g of each honey sample were dissolved in 25 mL buffer solution using a 25 mL volumetric flask^78^.

#### The invertase assay procedure

For the samples, 0.5 mL of substrate solution was heated in a water bath at 40 °C for five minutes and 0.5 mL of honey solution was added, hand mixed and incubated for 20 min at 40 °C. Finally, 0.5 mL of the reaction termination solution was added and the absorbance was measured at the wavelength of 400 nm after 15 min (Sample Absorbance; A400)^78^.

For the blank, 0.5 mL of substrate solution was heated in a water bath at 40 °C for five minutes and 0.5 mL of the reaction termination solution was added, hand mixed and incubated for 20 min at 40 °C. Finally, 0.5 mL of the honey solution was added and the absorbance was measured at the wavelength of 400 nm after 15 min (Blank Absorbance; A blank). The invertase activity (U/Kg) was calculated by applying the below equation:

$$ Invertase \;activity \left( \frac{U}{Kg} \right) = 158.94{ }\left( {{\text{A}}400 - {\text{A blank}}} \right) $$

The activity of invertase (U/Kg) was divided by 7.345 to obtain the activity in invertase number (IN)^31^.

$$ Invertase \;activity \left( {IN} \right) = \frac{{{\text{Invertase activity }}\left( {\frac{{\text{U}}}{{{\text{Kg}}}}} \right)}}{7.345} $$

### Statistical analysis

The results of this study were analyzed using the Statistical Package of Social Sciences (SPSS). Namely, the t-test and Analysis of Variance (ANOVA test) were used for the comparison between the mean values of the studied parameters. The significance was set at the level ≤ 0.05. Moreover, the agglomerative hierarchical clustering and multiple linear regression analysis were carried out to examine the efficiency of the studied parameters in predicting the floral and geographical origins of the honey samples.

## Data Availability

The datasets generated and/or analyzed during the current study are available from the corresponding author on request.
